# Pedabarography May Play a Role in Foot Plantar Scanning in Acromegaly

**DOI:** 10.1155/2022/9882896

**Published:** 2022-05-17

**Authors:** Tulay Omma, Azize Reda Tunc, Sevde Nur Fırat, Isılay Taskaldıran, Cavit Culha, Nese Ersoz Gulcelik

**Affiliations:** ^1^Department of Endocrinology and Metabolism, University of Health Sciences, Ankara Training and Research Hospital, 06230 Ankara, Turkey; ^2^Department of Physiotherapy, Lokman Hekim University, Faculty of Health Sciences, 06510 Ankara, Turkey; ^3^Department of Endocrinology and Metabolism, University of Health Sciences, Gulhane Training and Research Hospital, 06010 Ankara, Turkey

## Abstract

**Aims:**

Acromegaly is associated with symptoms in many organs, including the heart, colon, skin, bones, and many joints. Patients with long-term treatment or biochemical control still suffer from acromegaly arthropathy (AA). Primarily, the weight-bearing joints of the lower extremity are affected and at last deformation emerges. The aim of this study is to detect the changes in the feet with pedabarography in patients with acromegaly.

**Materials and Methods:**

Nineteen patients with acromegaly (4 males and 15 females) and 13 healthy controls (1 male and 12 females) were included in the study (*p*=0.31). There was no difference between acromegaly patients and controls in terms of gender, age, and BMI; median age and BMI were (54 (20–67) vs. 52 (30–58), *p*=0.85) and (32.5 (20.3–42.7) vs. 29.5 (22.4–38.6), *p*=0.93), respectively. Static plantar pressures of bilateral foot of all participants in the standing position were measured by pedabarography.

**Results:**

In pedabarographic analysis, there were only significant difference in rearfoot surface right and rearfoot surface left (*p*=0.04 and *p*=0.01), respectively. The mean of the right rearfoot surface (43.5 cm^2^ vs. 36.6 cm^2^) and the mean of the left rear foot surface were higher than the controls (47.4 cm^2^ vs. 40.2 cm^2^). Forefoot surface, forefoot load, forefoot weight ratio, rearfoot load, total foot surface, total load, total peak pressure, and total average pressure were higher in left foot in both groups, but there was no difference between the two groups.

**Conclusion:**

In our study, there was a significant difference between acromegaly patients and healthy controls, only on the right rarefoot surface and the left rarefoot surface, and was higher on the left in both groups. These patients often experience changes in the hindfoot and heel, and foot surface area and pressure distribution may vary. Early diagnosis and proper treatment of the disease can prevent the development of complications and improve the quality of life. Foot scanning using pedabarography in the management of AA is a useful tool that can be used to manufacture customized orthopedic insoles and ergonomic shoe designs to prevent irreversible damage and reduce overload and lower extremity pain.

## 1. Introduction

Acromegaly is a chronic disease characterized by excessive secretion of growth hormone (GH), usually caused by a pituitary adenoma [1]. It has a prevalence of 2.8–13.7 cases and an incidence of 0.2–1.1 cases/100,000 per year [2]. The age at diagnosis ranges from 40 to 50 years and is delayed by about ten years [2]. Acromegaly is associated with increased morbidity and mortality and decreased quality of life [3].

Patients with acromegaly usually present with acral bone growth resulting in frontal protrusion, increased hand and foot size, prognathism, and widening between lower incisors. It is also associated with manifestations of cardiovascular, gastrointestinal, skeletal system, and skin [4]. Acromegaly arthropathy (AA) is a common disease seen in 50–70% of patients and affects both axial and peripheral bones [5]. The most commonly involved peripheral joint is the knee. This is followed by joints of shoulder, hip, ankle, elbow, and hand [6]. Although joint stiffness and swelling are common, AA is generally noninflammatory. The hands and feet become sponge-like and swollen, and this is particularly evident in the heel pads [7].

Because acromegaly joint disease has common features with primary osteoarthritis (OA), it is often considered a degenerative joint disease. However, despite the narrowing of the joint space due to cartilage loss in osteoarthrosis, the enlargement of the joint space due to cartilage hypertrophy accompanied by severe osteophytosis in AA is the most important difference between them [6]. Enlargement of joint spaces and periarticular soft tissue hypertrophy are seen on radiographic images. This phase can be partially reversed by the normalization of GH and insulin such as growth-factor 1 (IGF-I). However, the persistent excess of GH/IGF-I causes degradation of joint structure through the repetition of intra-articular trauma and ultimately leads to irreversible disproportionate proliferation of regenerative fibrocartilage, scar, cyst, and osteophyte formation [8]. At this stage, the reduction in GH/IGF-I has limited effect on improvement of joint symptoms. Although the pathophysiology of joint destruction is not fully known, disorders related to the musculoskeletal system are responsible for actual functional disablement. Despite long-term treatment or biochemical control, patients still continue to suffer from AA [9].

AA is mostly evaluated by clinical and radiographic abnormalities. Although several clinical methods have been used to assess stiffness, pain, joint function, and quality of life, these have only been validated in primary OA patients. Early diagnosis of acromegaly can improve quality of life by reducing the risk of bone and joint disease. With the treatment of acromegaly, structural changes such as cartilage hypertrophy and joint thickening are partially reversed [10].

The foot has a structure that can easily adapt to both weight changes and changes in the printed surface. Chronic enlargement of soft tissue and bone can cause both somatic and gait disturbances. In addition, gait may be impaired due to biomechanical changes in acromegaly altering posture and balance, deformities may develop especially in the feet, and this may be irreversible. Foot deformities also change plantar pressure, and it has been proven that pressure characteristics differ according to foot deformities [11]. Knowing the pressure distributions and contact surfaces on the soles provides information about both the structure and function of the foot. The foot analysis of these patients can provide patient-specific ergonomic footwear designs and prevent the progression of deformities.

Pedobarography examines interactions between foot and support surface and also performs biomechanical analysis of gait and posture. Static pedobarography analyzes plantar pressure while standing, and dynamic pedobarography provides data during activities. Plantar foot pressure measurements can provide information about posture control [12]. Its technique is accurate, fast, and easy to apply and harmless, and its ability to be used in both clinical and research settings is promising. However, the use of these devices in the clinic is not yet common.

In the long run, irreversible changes in posture, balance, gait, and plantar pressure occur in the joints, especially in the foot joints, and reduce the quality of life in patients with acromegaly. Hitherto is the bony feature of acromegaly has only been observed from radiographic images, mostly in a few studies using ultrasonography or magnetic resonance imaging (MRI) for joints of the hands and a few studies using x-rays and ultrasound for foot changes [13–15]. None of these methods are standardized in the follow-up protocol. We support the hypothesis that the foot surface and pressures may change by time in patients with acromegaly. The aim of this study is to evaluate the plantar changes in patients with acromegaly assessed by pedobarography and compare them with healthy subjects.

## 2. Materials and Methods

### 2.1. Study Population

Nineteen acromegalic adult patients (4 males and 15 females; 20–67 years aged) and thirteen healthy subjects (1 male and 12 female; 30–58 years aged) admitted to the endocrinology clinic of Ankara Training and Research Hospital were enrolled in this cross-sectional study. The diagnosis of acromegaly was confirmed by clinical, hormonal, and radiological data. The control group included volunteers who had no previous foot or ankle problems and whose gender, age, and body mass index were matched with acromegaly patients.

Those with foot deformities such as pes cavus, pes planus, hallux valgus, and a history of foot surgery or major trauma and those with rheumatic diseases or nerve entrapment lesions such as mortone neuroma were excluded from the study.

Demographic and clinical features of acromegaly patients and control group were recorded into a predefined protocol. All the patients underwent analysis with a static pedobarogram to evaluate plantar pressure changes and compared with the healthy control group.

Ethics approval was obtained from the Ankara Numune Training and Research Hospital Ethics Committee. The research protocol is in line with the 2000 Helsinki Declaration and written informed consent was obtained from all participants.

### 2.2. Determination of Serum GH and IGF-1 Levels

All serum samples were taken in the early morning hours with a 12-hour fast. Serum GH concentrations were analyzed by electrochemiluminescence (ECLIA, Cobas e601) with a detection limit of 0.01 *μ*g/L. The interassay CV was %1.9, while the intra-assay CV was % 3.0. Serum IGF-1 levels were analyzed by RIA (Berthold gama gaunter). The intra-assay CV was 3.3%, while the interassay CV was 7.2%.

Patients with normal IGF-1 values according to age- and gender-specific levels and a spot or suppressible GH values < 1 *μ*g/L were classified as acromegaly in remission.

### 2.3. Determination of Static Plantar Variables

Plantar pressure during static standing position was measured using the Footscan® 3D System (RScan International, Olen, Belgium). The system includes pressure-sensing platform, power unit, camera, monitor, and printer connections and connected to the computer system. The pressure platform contained 16384 resistive sensors arranged in 200 cm × 40 cm active sensor area with dimensions of 1800 cm × 40 cm and with a data acquisition frequency 100 Hz. The Footscan® 3D system was calibrated before each test session, according to the manufacturer's manual. By software analysis, the pressure distribution of low and high pressure in the form of specific “hot” and “cold” zones is determined topographically. Using software support, the device we used in this study evaluated the foot by dividing it into the forefoot and rearfoot regions. Plantar pressure was determined using the peak pressure (kPa), force (Ns), and area (cm) parameters.

Prior to data collection, at least 3 statistical posture rehearsals were completed on the pressure platform to ensure conditioning. They were asked to place their feet in the designated area on the platform. After maintaining a static posture on the platform, they were asked to look at the object placed at eye level and placed on the wall in front of them and measurements provided. In the static pedobarographic evaluation of the foot, maximal pressure values in the forefoot and hindfoot, total pressure in the foot, percentages of total pressure on the front/rear part of the foot, and total contact area and percentage share values of the total contact area to the fore and hind foot are obtained.

## 3. Statistical Analysis

Statistical analysis was performed using the 21.0 version of the Statistical Package for Social Sciences (SPSS) program (SPSS Inc., Chicago, IL). The Kolmogorov–Smirnov test helped us to check whether continuous variables showed a normal distribution. Normally distributed continuous variables are defined as mean ± SD, while nonnormally distributed parameters are reported as medians (minimum-maximum). Comparison between groups of continuous variables was carried out using Student's *t*-test or Mann–Whitney *U* test. The Chi-square test was used to compare groups for categorical variables. *p* value <0.05 was considered significant in all statistical analyzes.

## 4. Results

**Figure 1:**
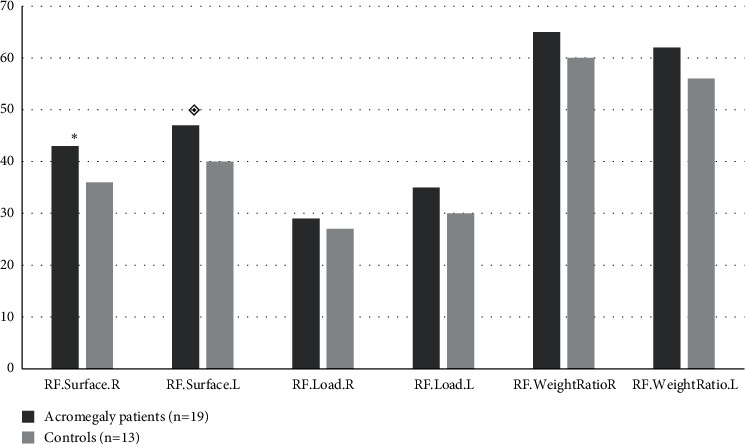
Analysis of the rearfoot results. ^*∗*^*p*=0.04, statistical significance for RF.Surface R. *p*=0.01, statistical significance for RF.Surface L. RF.Surface.R, rearfoot surface right, cm^2^; RF.Surface.L, rearfoot surface left, cm^2^; RF.Load.R, rearfoot load right, %; RF.Load.L, rearfoot load left, %; RF.WeightRatio.R, rearfoot weight ratio right, %; RF.WeightRatio.Left, rearfoot weight ratio left, %.

**Figure 2:**
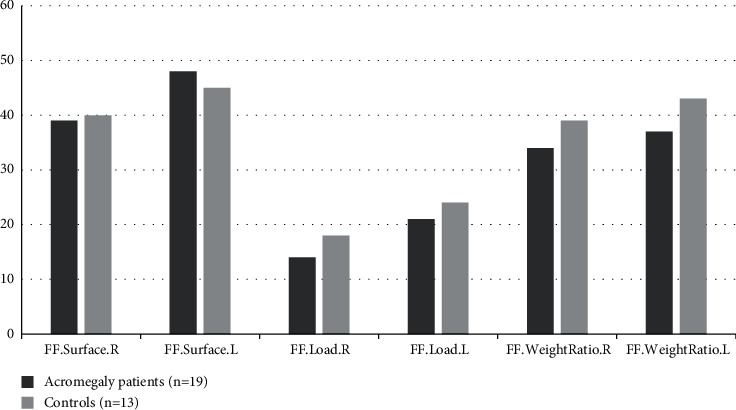
Analysis of forefoot results. FF.Surface.R, forefoot surface right, cm^2^; FF.Surface.L, forefoot surface left, cm^2^; FF.Load.R, forefoot load right, %; FF.Load.L, forefoot load left, %; FF.WeightRatio.R, forefoot weight ratio right, %; FF.WeightRatio.Left, forefoot weight ratio left, %.

**Figure 3:**
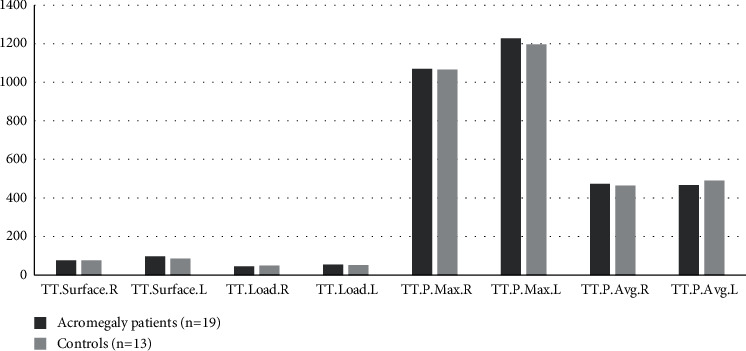
Analysis of total foot results. TT.Surface.R, total surface right, cm^2^; TT.Surface.L, total surface left, cm^2^; TT.Load.R, total load right, %; TT.Load.L, total load left, %; TT.P.Max.R, total pressure maximum right, gr/cm^2^; TT.P.Max.L, total pressure maximum left, gr/cm^2^; YT.P.Avg.R, total pressure average right, gr/cm^2^; TT.P.Avg.L; total pressure average left, gr/cm^2^.

The gender (*p*=0.31), ages, and body mass indexes were matched between acromegaly patients and control group; median age and BMI were (54 (20–67) vs. 52 (30–58), *p*=0.85) and (32.5 (20.3–42.7) vs. 29.5 (22.4–38.6), *p*=0.93), respectively. The main characteristics of patients with acromegaly are presented in Table 1. In pedobarographic analysis there were only significant difference in rearfoot surface right and rearfoot surface left, *p*=0.04 and *p*=0.01, respectively (Figure 1). In acromegaly patients, both the mean of the right hind foot surface was higher than the controls (43.5 cm^2^ vs. 36.6 cm^2^) and the mean of the left hind foot surface was higher than the controls (47.4 cm^2^ vs. 40.2 cm^2^). Forefoot surface (48.94 cm^2^ vs 45.31 cm^2^, *p*=0.65), forefoot load (21% vs. 24.2%, *p*=0.43), forefoot weight ratio (37.1% vs. 43.1%, *p*=0.32) (Figure 2), rearfoot load (35.2% vs. 30.7%, *p*=0.18), total foot surface (96.21 cm^2^ vs. 85.46 cm^2^, *p*=0.31), total load (56.2% vs. 54.85%, *p*=0.72), total peak pressure (1228 gr/cm^2^ vs. 1196.1 gr/cm^2^, *p*=0.82), and total average pressure (529.5 gr/cm^2^ vs. 515.6 gr/cm^2^, *p*=0.81) were higher in left foot in both groups, but there was no difference between the two groups (Figure 3).

## 5. Discussion

In this study, we inspected the static barefoot plantar pressure changes in acromegaly patients. The present study is one of the first research studies to address plantar statics using pedobarography in this patient group. In this study, we found that there was a difference in the surface of the right rear foot and the surface of the left rear foot in patients with acromegaly compared to healthy controls. Also, the mean of rearfoot surface right and left in acromegaly patients was higher than controls. Although there was no difference between the two groups, the total surface area was higher in the acromegaly group in the left foot, while it was similar in the right foot. Although weight matched between groups, the total load on the right foot was higher in the control group, while the total load on the left foot was higher in the acromegaly group. Peak pressure and average pressure in both forefoot and rearfoot were higher in the acromegaly group.

Because of the insidious nature of the disease, the diagnosis of acromegaly is significantly delayed. The musculoskeletal manifestations of the syndrome are common, and almost all patients develop symptoms or signs of arthropathy, but these symptoms have been poorly studied [16]. On joint radiographs, a decrease in both weight-bearing and nonweight-bearing joint spaces is observed in the first 5–10 years after treatment, which is a long enough period for the development of AA. Sometimes early signs of joint involvement can be observed in patients with a short duration of disease [14]. The main causes of morbidity in acromegaly are arthropathy and enthesopathy [17]. The first step of AA is considered to be cartilage and soft tissue hypertrophy. The pathogenesis of arthropathy comprises both GH/IGF-I excess and secondary degenerative changes. DNA synthesis, cell replication, proteoglycan, and glycosaminoglycan synthesis are stimulated in articular chondrocytes by GH and locally produced IGF-I [18].

Currently, only IGF-1 and GH blood values are recommended for acromegaly to detect possible disease activity and to evaluate treatment outcomes or possible complications during the follow-up period. However, these values may not adequately reflect disease activity in peripheral tissues, especially during medical treatment. Since the effectiveness of treatment modalities such as humidity and infection control, hyperbaric oxygen therapy, negative pressure wound therapy, and debridement, which are frequently used in diabetic foot ulcers, are not always successful, as a new treatment modality adding Lactiplantibacillus plantarum ATCC 10241 cultures on venous ulcers has been tried, and encouraging positive results have been obtained [19]. It is also possible to compare different treatment options, and their effects on soft tissues in patients with acromegaly.

In acromegaly patients, the shoe size showing the increase in the plantar surface grows over time. Sponginess and puffiness hands and feet (predominantly due to soft tissue swelling) are present in the vast majority of acromegalic patients; this is particularly noticeable in heel pads. The vast majority of acromegalic patients have spongy and swollen hands and feet (mostly due to soft tissue swelling); this is particularly evident in heel pads. This finding can be made by clinical examination or imaging studies and is quite evident.

Radiogram abnormalities of the hip, knee, and hand are also found in more than 50% of patients [20]. Particularly, in peripheral joints, the early radiological finding is joint space enlargement, while features such as narrowing of joint spaces, osteophytosis, and other osteoarthritis are seen in long-term disease [18]. In a study by Bukhman and Avakian, X-ray examination of foot soft tissues and measurement of foot soft tissue thickness (FSTT) were performed in patients with acromegaly. They suggested that elevated FSTT over 22 mm and changes in foot soft tissues are characteristic for acromegaly, and this value can serve as an auxiliary sign for the diagnosis of early stages of disease as well as for the assessment of a degree of activity and efficacy of long-term results of therapy of acromegaly [21].

Colao et al. demonstrated the usefulness of joint ultrasound in measuring cartilage thickness and monitoring the effect of octreotide therapy [22]. They also showed improvement in enthesopathy, with significant reduction in heel tendon size. However, both radiographic and ultrasonographic examinations fail to measure foot plantar pressures. However, with the pedabarography technique, foot sole surface and pressure changes can be detected objectively and earlier, and there may be some benefits to using barefoot measurements to help create special insoles to prevent arthropathy and enthesopathy that may occur in the foot. In our study there was no difference between plantar pressures, but this is probably due to the small sample size. However, acromegaly patients generally had a tendency to pronation. This should be taken into account as abnormal pronation in the foot can cause problems such as increased flexibility, impaired load distribution, hallux valgus and heel spurs, and postural disorders involving the leg, knee, hip, and lower back over time.

There is only one study in the literature by Sendur et al. in which pedabarography was used in acromegaly patients [23]. In their study, they could not find any significant difference between active and remission acromegaly patients in terms of static foot scan parameters. In our study, consistent with them and previous radiological studies, the averages of the right and left hindfoot surfaces were higher in acromegaly patients compared to controls, and there were statistical differences between the two groups. Although there was no difference between the two groups, the total surface area was higher in the left foot in the acromegaly group, but similar in the right foot. Furthermore, in the study of Sendur et al., mean pressure on the foot was higher in patients with acromegaly, whereas total load, total peak pressure, and total mean pressure in the left foot were higher in both groups in our study, but there was no difference between the two groups.

Whether AA can be reversed by controlling GH and IGF-I levels is still being questioned. Significant structural changes in the joints and limited restorative ability of chondrocytes seem to prevent healing in AA. However, surgery or treatment with somatostatin analogues has been shown to improve the signs and symptoms of AA [24].

However, in the study of Colao A et al. it was observed that neither cure nor octreotide treatment was sufficient for cartilage thickness to return to similar values with healthy subjects [14]. However, in another study of the same group, it was shown that thickening of shoulder, wrist, and knee cartilages and heel tendons was significantly reduced with lanreotide treatment for 12 months in all patients [22]. It is very important to take care of chiropody before irreversible changes occur. Despite the advanced current findings in the literature such as artificial intelligence assisted thermal change index-based foot thermogram for the detection of plantar disorders, there are no routine recommendations in the guidelines for the follow-up of foot complications in acromegaly [25].

First of all, although the prevalence of inflammatory rheumatic diseases is low in these patients, its exclusion and accurate diagnosis of AA is necessary. Since there are no sensitive biomarkers to manage AA other than monitoring disease activity with IGF-1 and GH testing, it is usually followed according to the clinical course of joint problems. In general, when joint problems become serious, consultation with a rheumatologist or orthopedic surgeon is considered to evaluate possible treatment options. However, the role of the physiotherapist in this regard has not been investigated yet. The findings of present study underline that AA is a distinct clinical entity with its own characteristics. Future studies are needed to determine whether acromegaly-specific interventions such as physiotherapy could be useful in the management of AA.

The type of material to be used in the manufacture of orthopedic insoles is selected according to the clinical picture, pedobarography findings, and the need to reduce the load on certain parts of the foot. Therefore, in the diagnosis and follow-up of AA, footscan is a useful tool that can be used to prevent irreversible damage to the foot. So, the pedabarographic examination results enable the selection of suitable insoles that help reduce overload and lower extremity pain and back pain.

This study has a few limitations. Firstly, the number of patients in this pilot study is relatively small. Studies involving more patients are needed to reach more accurate results. Secondly, the acromegaly group in the study was quite heterogeneous as it included both active and remitting patients. We did not consider foot types, which is another factor that will affect pressure distribution.

## 6. Conclusion

Acromegaly is usually a slow progressing disorder. The foot changes that mostly skipped can cause imbalance and falls by time. Early diagnosis and proper treatment of the disease can prevent the development of complications and improve the quality of life. These patients often experience changes in the hindfoot and heel, and foot surface area and pressure distribution may vary. In our study, by pedabarographic analysis, there was a significant difference between acromegaly patients and healthy controls, only on the right rarefoot surface and the left rarefoot surface, and was higher on the left in both groups. However, rarefoot load, forefoot surface, and forefoot load were also higher on the left in both groups, but there was no difference between the groups. Foot scanning using pedabarography in the management of AA is a useful tool that can be used to produce customized orthopedic insoles and ergonomic shoe designs to prevent irreversible damage, reduce overload, and lower extremity pain and back pain.

## Figures and Tables

**Table 1:** Characteristics of acromegaly patients.

Age (years)^a^	54 (20–67)
Gender	15 females and 4 males
Duration of illness (years)^a^	6 (0–20)
Body mass index (kg/m^2^)^b^	32.14 ± 5.76
GH (*μ*g/L)^a^	0.96 (0.03–4.2)
IGF-I (ng/ml)^b^	153.83 ± 143.24
History of operation	15 patients 1 time and 4 patients twice
History of radiotherapy	14 patients never and 5 patients 1 time
Remission	14 patients are in remission and 5 are not
Arthralgia	17 patients have not and 2 have

## Data Availability

The data used to support the findings of this study are restricted by the Ankara Numune Training and Research Hospital Ethics Committee in order to protect Patients Privacy and the rights of the private center that owns the pedabarography device. The data used to support the findings of the study can be obtained from the corresponding author upon request, for researchers who meet the criteria for access to confidential data.

## References

[1] Melmed S. (2009). Acromegaly pathogenesis and treatment. *Journal of Clinical Investigation*.

[2] Daly A. F., Rixhon M., Adam C., Dempegioti A., Tichomirowa M. A., Beckers A. (2006). High prevalence of pituitary adenomas: a cross-sectional study in the Province of Liège, Belgium. *Journal of Clinical Endocrinology and Metabolism*.

[3] Dekkers O. M., Biermasz N. R., Pereira A. M., Romijn J. A., Vandenbroucke J. P. (2008). Mortality in acromegaly: a metaanalysis. *Journal of Clinical Endocrinology and Metabolism*.

[4] Lamberts S. W. J., van der Lely A. J., de Herder W. W. (1995). Clinical and medical diagnosis of acromegaly. *Metabolism*.

[5] Barkan A. L. (2001). *Pituitary*.

[6] Wassenaar M. J. E., Biermasz N. R., Bijsterbosch J. (2011). Arthropathy in long-term cured acromeagaly is characterised by osteophytes without joint space narrowing: a comparison with generalised osteoarthritis. *Annals of the Rheumatic Diseases*.

[7] Colao A., Pivonello R., Scarpa R., Vallone G., Ruosi C., Lombardi G. (2005). The acromegalic arthropathy. *Journal of Endocrinological Investigation*.

[8] Okazaki K., Jingushi S., Ikenoue T. (1999). Expression of insulin-like growth factor I messenger ribonucleic acid in developing osteophytes in murine experimental osteoarthritis and in rats inoculated with growth hormone-secreting tumor. *Endocrinology*.

[9] Romijn J. A. (2013). Acromegalic arthropathy: current perspectives. *Endocrine*.

[10] Gadelha M. R., Kasuki L., Lim D. S. T., Fleseriu M. (2019). Systemic complications of acromegaly and the impact of the current treatment landscape: an update. *Endocrine Reviews*.

[11] Zhang Bo, Lu Q. (2020). A current review of foot disorder and plantar pressure alternation in the elderly. *Physical Activity and Health*.

[12] Orlin M. N., McPoil T. G. (2000). Plantar pressure assessment. *Physical Therapy*.

[13] Biermasz N. R., van ‘t Klooster R., Wassenaar M. J. E. (2012). Automated image analysis of hand radiographs reveals widened joint spaces in patients with long-term control of acromegaly: relation to disease activity and symptoms. *European Journal of Endocrinology*.

[14] Colao A., Marzullo P., Vallone G. (1998). Reversibility of joint thickening in acromegalic patients: an ultrasonography study. *Journal of Clinical Endocrinology and Metabolism*.

[15] Lieberman S. A., Björkengren A. G., Hoffman A. R. (1992). Rheumatologic and skeletal changes in acromegaly. *Endocrinology and Metabolism Clinics of North America*.

[16] Tagliafico A., Resmini E., Ferone D., Martinoli C. (2011). Musculoskeletal complications of acromegaly: what radiologists should know about early manifestations. *La Radiologia Medica*.

[17] Colao A., Lombardi G. (1998). Growth-hormone and prolactin excess. *Lancet*.

[18] Barkan A. (1997). Acromegalic arthropathy and sleep apnea. *Journal of Endocrinology*.

[19] Argañaraz Aybar J. N., Ortiz Mayor S., Olea L. (2022). Topical administration of Lactiplantibacillus plantarum accelerates the healing of chronic diabetic foot ulcers through modifications of infection, angiogenesis, macrophage Phenotype and neutrophil response. *Microorganisms*.

[20] Layton M. W., Fudman E. J., Barkan A., Braunstein E. M., Fox I. H. (1988). Acromegalic arthropathy. *Arthritis and Rheumatism*.

[21] Bukhman A. I., Avakian M. R. (1989). The soft tissues of the foot in acromegaly. *Problemy Endokrinologii*.

[22] Colao A., Marzullo P., Vallone G. (1999). Ultrasonographic evidence of joint thickening reversibility in acromegalic patients treated with lanreotide for 12 months. *Clinical Endocrinology*.

[23] Sendur S. N., Oguz S., Dagdelen S., Erbas T. (2019). Assessment of static and dynamic plantar data of patients with acromegaly. *Pituitary*.

[24] Gorden P., Comi R. J., Maton P. N., Go V. L. (1989). Somatostatin and somatostatin analogue (SMS 201-995) in treatment of hormone-secreting tumors of the pituitary and gastrointestinal tract and non-neoplastic diseases of the gut. *Annals of Internal Medicine*.

[25] Khandakar A., Chowdhury M. E. H., Reaz M. B. I. (2022). Thermal change index-based diabetic foot thermogram image classification using machine learning techniques. *Sensors*.

